# Applying Queuing Theory and Mixed Integer Programming to Blood Center Nursing Schedules of a Large Hospital in China

**DOI:** 10.1155/2020/9373942

**Published:** 2020-07-01

**Authors:** Li Luo, Xiaofei Liu, Xinyuan Cui, Yuanjun Cheng, Xinzhu Yu, Yue Li, Li Jiang, Mingying Tan

**Affiliations:** ^1^Business School, Sichuan University, Chengdu, China; ^2^Sichuan Provincial People's Hospital, Chengdu, China; ^3^West China Hospital, Chengdu, China

## Abstract

Blood centers in large hospitals in China are facing serious problems, including complex patient queues and inflexible nursing schedules. This study is aimed at developing a flexible scheduling method for blood center nurses. By systematically analyzing the constraints that affect scheduling, a flexible scheduling model is established based on queuing theory and mixed integer programming. This combined model can reasonably determine the number of nurses required during a given working period and flexibly arrange nursing schedules while ensuring sufficient rest periods for individual nurses. Results of numerical studies conducted using data from a large hospital in China show a significant improvement in patient waiting time performance metrics over the hospital's current practice. In addition, the nurses' workloads and rest periods are well balanced, indicating that the proposed method can effectively and flexibly arrange nursing shifts in blood centers.

## 1. Introduction

Healthcare resources in China are currently under pressure because of high demand and low supply, with 20% of the world's population having to cope with no more than 3% of global healthcare resources [1]. Human staff is a vital medical resource; in particular, nursing staff considerably impact clinical outcomes. Nurse scheduling is presently the most demanding scheduling problem affecting hospital personnel on a daily basis [2]. Many hospital nursing managers still rely on manual scheduling to create nurses' shift patterns. Although experienced managers can easily perform this task in small hospitals, it can become a very difficult and time-consuming task in large hospitals. In addition, fairly distributing the workload among nurses can directly affect their job satisfaction.

The number of patients arriving at different times in the blood center substantially fluctuates. Such fluctuations often cause two unfortunate situations: (1) the service's peak capacity is insufficient to meet the demand, causing long queues, and (2) the service's capacity during quiet periods is much higher than the demand, wasting human resources. In addition, heavy workloads, high pressure, and lack of necessary rest periods make it easy for blood collection nurses to get stressed and make mistakes, further affecting the quality of service. Although many service industries have introduced part-time workers to ease work pressure during peak hours, hospitals around the world rarely employ such staff [3]. We introduce part-time nurses to ease the stress on regular nurses during peak hours. In addition, we have reduced the number of nurses working in low hours needed to avoid waste of resources.

The most important aspect of blood center nurse scheduling is to accurately calculate the demand for nurses. How to properly determine the number of nurses who need to work for providing the most convenient medical services for patients? The operational research model offers a systematic approach to problem solving and allows for the characterisation of activities of an existing system using mathematical modelling. Saville et al. believed that the introduction of operational research techniques into mainstream nurse research can enhance decision-making on nurse staffing and discussed the application of these techniques in the medical field [4]. One approach, based on mathematical models, that successfully addresses problems in healthcare systems is the use of queueing models [5]. Izady and Worthington proposed an iterative scheme that uses an infinite server network, square root staffing, and simulation to set the minimum medical staffing levels required to achieve government goals [6]. Jahn et al. used queuing theory to simulate patient arrivals and assess the resulting economic effects, proving that queuing theory produced better results [7]. According to the fact that the patients' arrival rate is dynamic and random, Liu et al. and Lin et al. used the M/M/C queuing theory to estimate the patient's waiting time in the system of dynamic demand and then constructed the mixed integer programming model for EMR scheduling to obtain a flexible shifting scheme [8, 9]. Chen and Ping used a queuing theory model to study the queuing system and its effects on a hospital's blood collection hall and determined the optimal number of nursing staff required to meet the needs of both patients and the staff themselves [10].

Although some hospitals may use queuing theory to create nursing schedules, this theory only calculates the number of nurses required to prevent patients' queuing for a long time and does not consider the broader issues involved in creating suitable nursing schedules. The scheduling problem of medical staff is a combinatorial optimization problem. Manual scheduling is tedious and time-consuming; therefore, the nurse rostering problem (NRP), also known as the nurse scheduling problem (NSP), has attracted considerable research attention.

In these research studies, some soft and hard constraints are considered, including the policies of the state, rules of the hospital, and personal needs of doctors and nurses [11]. The 0-1 integer programming model, mixed integer programming model, and the goal programming model are constructed, and the exact algorithm and heuristic algorithm are used to solve them [12, 13]. In order to obtain the scientific and reasonable scheduling shifts, Tan et al. consider various management rules in a hospital, physicians' personal preferences, and the time requirements of their personal learning and living and take the minimum deviation variables from the soft constraints as the objective function to construct a mixed integer programming model with the doctor group as the scheduling unit [14]. In addition, some scholars use heuristic algorithms to solve the problem of nurses' scheduling. Wu et al. proposed a particle swarm optimization method to solve highly complex NSPs to generate nursing schedules that meet all requirements and consider fairness [15]. Lin designed an adaptive scheduling heuristic algorithm that reduces the patients' waiting times [16]. Zhong et al. proposed a two-stage heuristic algorithm for nurse scheduling to achieve fairness and flexibility goals [17].

This study is aimed at nurse scheduling models for accommodating different patient needs as well as for satisfying the working rules and regulations, thereby generating nursing rosters that adhere to the hospital fairness requirements. Then, we introduce part-time nurses to ease the stress on regular nurses during peak hours. A queuing model is used herein to calculate the labor demands for each period during the day. This model calculates the minimum staffing levels required for each period to meet the hospital's needs. Based on these minimum staffing requirements, a mixed integer programming (MIP) model is proposed to determine the most effective nursing roster. The proposed model is numerically investigated to validate the model's feasibility.

## 2. Materials and Methods

### 2.1. Study Hospital

Our survey of a hospital in Chengdu revealed that the blood center, an important outpatient service, has always been unable to satisfy the increasing demand because of the increasing number of patients; this results in long queues and affects patient satisfaction. Patients wait for an average of 35 min and maximum up to 67 min. Because of the long waiting time, patients have to simultaneously wait for testing their blood.

**Figure 1:**
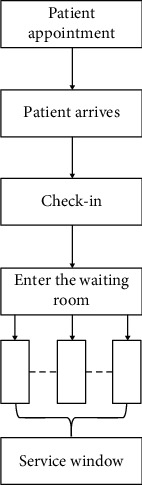
Patient consultation flow chart.

**Figure 2:**
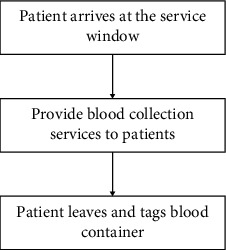
Doctor service flow chart.

As we all know, each nurse has his own role in a department to complete a series of operations. However, our research is limited to a blood collection center. In the blood collection center, the nurse has only one operation for blood collection. Some things like supporting employees and preparing records are all done by smart devices. Figures 1 and 2 provide the flow chart of patient consultation and the flow chart of doctor service, respectively. Patient appointments can also be made through online appointments or offline machines. When the patient registers, the patient only needs to swipe the medical card to record all the information. As for the nurse's job, only blood collection and labeling of blood containers are required. In addition, the labeling process is assisted by intelligent machines.

### 2.2. Datasets

Our models are based on a dataset that includes 119302 patient blood collection records collected from hospital H's blood center between October 9, 2017, and January 31, 2018. Herein, work begins at 7:00 a.m. and finishes at 5:00 p.m. After processing and analyzing these data using R, we calculated the patient arrival rates and average service time. Then, we used a goodness-of-fit test to determine that the patient arrival rates obeyed the Poisson distribution.

### 2.3. Methods

The proposed models involve two stages. First, we calculate the most reasonable number of staff by a queuing model; then, we reasonably arrange their schedules by a mixed integer programming model.

#### 2.3.1. Queuing Model

The patient arrival rates are subject to the Poisson distribution; therefore, queuing theory can be used to calculate the minimum number of staff required to meet the demand. Specifically, we propose the following solution to find the most reasonable number of windows to open during each time shift.

The blood center's service quality requirements state that at least 95% of patients should leave the service within 15 min. We use queuing theory to calculate the optimal number of open windows and establish a queuing model. We use the following notation:

*c*: number of open windows in the blood center

*λ*: patient arrival rate

*μ*: average service rate

*ρ*: service intensity

*L*q: number of patients in the queue

*W*q: average patient waiting time

*t*q: hospital's required maximum average patient waiting time

*t*: hospital's required maximum average time that patients stay in the center

*p*q: probability of achieving the average waiting time requirement

*P*: probability of achieving the average staying time requirement

*L*: average number of patients in the queuing system

*W*: average time patients stay in the blood center

*u*: average utilization rate of the blood center

$\overset{-}{c}$: average number of busy windows

The number of open windows in the blood center must be a positive integer, and this value cannot be analytically obtained. Therefore, we designed the following algorithm to calculate the value of the decision variable.

**Step 1 .**

For the queuing process to reach a steady state, *c* = [*λ*/*μ*] (an integer not exceeding [*λ*/*μ*]).

**Step 2 .**

If *W*q ≤ 15 min, then terminate and return to *c* as the minimum number of open windows required; otherwise, go to Step 3.

**Step 3 .**

*c* = *c* + 1 and go to Step 2.

The queuing model is represented as follows:

(1) $$\begin{array}{c} \min c, \end{array}$$

subject to

(2) $$\begin{array}{c} P\left\{W_{\text{q}}\leq t_{\text{q}}\right\}=1-P\left\{W_{\text{q}}>t_{\text{q}}\right\}=1-P_{\text{q}}>p_{\text{q}}m, \end{array}$$

(3) $$\begin{array}{c} P\left\{W\leq t\right\}=1-P\left\{W>t\right\}=1-P\geq p, \end{array}$$

(4) $$\begin{array}{c} c\in Z^{+}, \end{array}$$

(5) $$\begin{array}{c} P_{\text{q}}=P\left\{W_{\text{q}}>t_{\text{q}}\right\}=\left(1-P\left\{W_{\text{q}}=0\right\}\right)e^{-c\mu \left(1-\rho \right)t\text{q}}, \end{array}$$

(6) $$\begin{array}{c} P\left\{W_{\text{q}}=0\right\}=\overset{c-1}{\underset{0}{\sum }}p_{n}, \end{array}$$

(7) $$\begin{array}{c} P=P\left\{W>t\right\}=e^{-\mu t}\left[1+\frac{p0{\left(\lambda /\mu \right)}^{c}}{c!\left(1-\left(\lambda /c\mu \right)\right)}\left(\frac{1-e^{-\mu t\left(c-1-\left(\lambda /\mu \right)\right)}}{c-1-\left(\lambda /\mu \right)}\right)\right], \end{array}$$

(8) $$\begin{array}{c} p0={\left[\overset{c-1}{\underset{n=0}{\sum }}\frac{{\left(\lambda /\mu \right)}^{n}}{n!}+\frac{{\left(\lambda /\mu \right)}^{c}}{c!}\left(\frac{1}{1-\left(\lambda /c\mu \right)}\right)\right]}^{-1},\;\text{if}\frac{\lambda }{c\mu }<1, \end{array}$$

(9) $$\begin{array}{c} \begin{array}{c} p_{0}=\left\{{}_{\frac{{\left(\lambda /\mu \right)}^{n}}{c!c^{n-c}}p_{0},\;n\geq c,}^{\frac{{\left(\lambda /\mu \right)}^{n}}{n!}p_{0},\;0\leq n\leq c,}\right. \end{array} \end{array}$$

(10) $$\begin{array}{c} L\text{q}=\frac{\rho {\left(\lambda /\mu \right)}^{c}}{c!{\left(1-\rho \right)}^{2}}p0,\;\rho =\frac{\lambda }{c\mu }, \end{array}$$

(11) $$\begin{array}{c} L=L\text{q}+\frac{\lambda }{\mu }, \end{array}$$

(12) $$\begin{array}{c} W\text{q}=\frac{L\text{q}}{\lambda }, \end{array}$$

(13) $$\begin{array}{c} W=\frac{L}{\lambda }, \end{array}$$

(14) $$\begin{array}{c} c\_=L-L\text{q}, \end{array}$$

(15) $$\begin{array}{c} u=\frac{\lambda }{c\mu }. \end{array}$$

Herein, the objective function (1) minimizes the number of open windows, constraint (2) represents the waiting time constraint, constraint (3) represents the staying time constraint, constraint (4) guarantees that *c* is an integer, constraints (5)–(9) calculate the number of open windows, and constraints (10)–(15) are various indicators used for evaluation.

#### 2.3.2. Nurse Scheduling Model

The above queuing model can be used to calculate the number of blood centers that need to be opened during each period of the day. Results provide the number of staff members required. Based on the results, an MIP model is established to generate daily rosters for the blood collection nurses. Creating flexible schedules for hospital blood collection nurses involves two main factors: the nurses have to receive sufficient rest periods and their working hours should be approximately the same.

The hospital introduces part-time nurses to alleviate the stress during peak hours and cover absences and lunch time. Most peak working hours are in the morning; therefore, these part-time nurses can only work during two time shifts in the morning. In actual scheduling scenarios, the need to combine many constraints with the number of blood collection nurses required during each time shift dramatically increases the complexity of the problem, making manual scheduling difficult to achieve.

We use the following additional notation:

*i*: full-time blood collection nurse identifier

*j*: part-time blood collection nurse identifier

*d*: day number in the roster period

*t*: period number in the day

*c*_*dt*_: minimum number of nurses required for time shift *t* on day *d* (obtained using the above queuing model)

nt: maximum number of time shifts for which a nurse can continuously work every day

nd_min_ and nd_max_: minimum and maximum number of days, respectively, for which a full-time nurse can work continuously during a given roster period

*z*_*id*_: work status of a full-time nurse *i* on day *d*

*m*_*jd*_: work status of a part-time nurse *j* on day *d*

*x*_*idt*_: indicates whether a full-time nurse *i* works during period *t* on day *d*

*y*_*jdt*_: indicates whether a part-time nurse *j* works during period *t* on day *d*

nj: maximum number of part-time nurses working on a given day

sumd_min_ and sumd_max_: minimum and maximum number of days worked by a full-time nurse during one roster period, respectively

The nurse scheduling model is represented as follows:

(16) $$\begin{array}{c} \min\left[z_{id}+\left(\overset{I}{\underset{i=1}{\sum }}x_{idt}+\overset{J}{\underset{j=1}{\sum }}y_{jdt}\right)\right], \end{array}$$

subject to

(17) $$\begin{array}{c} \overset{I}{\underset{i=1}{\sum }}x_{idt}+\overset{J}{\underset{j=1}{\sum }}y_{jdt}\geq c_{dt},\;\forall d,\forall t, \end{array}$$

(18) $$\begin{array}{c} -n\leq \overset{D}{\underset{d=1}{\sum }}\overset{T}{\underset{t=1}{\sum }}x_{i_{1}dt}-\overset{D}{\underset{d=1}{\sum }}\overset{T}{\underset{t=1}{\sum }}x_{i_{2}dt}\leq n,\;\forall i_{1},\forall i_{2},i_{1}\neq i_{2}, \end{array}$$

(19) $$\begin{array}{c} \overset{k+\text{nt}}{\underset{t=k}{\sum }}x_{idt}\leq \text{nt},\;\forall d,\forall i,\forall k=1,2,\cdots ,T-\text{nt}, \end{array}$$

(20) $$\begin{array}{c} \overset{k+\text{nt}}{\underset{t=k}{\sum }}y_{jdt}\leq \text{nt},\;\forall d,\forall j,\forall k=1,2,\cdots ,T-\text{nt}, \end{array}$$

(21) $$\begin{array}{c} {\text{nd}}_{\min}\leq \overset{k+\text{nd}}{\underset{d=k}{\sum }}z_{id}\leq {\text{nd}}_{\max},\;\forall i,\forall k=1,2,\cdots ,D-\text{nd}, \end{array}$$

(22) $$\begin{array}{c} M\cdot {mz}_{id}\geq \overset{T}{\underset{t=1}{\sum }}x_{jdt},\;\forall i,\forall d, \end{array}$$

(23) $$\begin{array}{c} M\cdot \left(z_{id}-1\right)+1\leq \overset{T}{\underset{t=1}{\sum }}x_{jdt},\;\forall i,\forall d, \end{array}$$

(24) $$\begin{array}{c} y_{idt}=0,\;\forall j,\forall d,t=T_{j}, \end{array}$$

(25) $$\begin{array}{c} {\text{sumd}}_{\min}\leq \overset{D}{\underset{d=1}{\sum }}z_{id}\leq {\text{sumd}}_{\max},\;\forall i, \end{array}$$

(26) $$\begin{array}{c} \overset{J}{\underset{j=1}{\sum }}m_{jd}\leq \text{nj},\;\forall d, \end{array}$$

(27) $$\begin{array}{c} M\cdot m_{id}\geq \overset{T}{\underset{t=1}{\sum }}y_{jdt},\;\forall j,\forall d, \end{array}$$

(28) $$\begin{array}{c} M\cdot \left(m_{id}-1\right)+1\leq \overset{T}{\underset{t=1}{\sum }}y_{jdt},\;\forall j,\forall d, \end{array}$$

(29) $$\begin{array}{c} x_{idt}=c\left(0,1\right),\;\forall i,\forall d,\forall t, \end{array}$$

Here, the objective function (16) minimizes the number of days worked by full-time nurses given that the total number of hours worked by all nurses is as small as possible. Constraint (17) indicates that a sufficient number of nurses must work daily to meet the patients' needs. Constraint (18) indicates that there should not be a considerable difference between the work periods of different full-time nurses in the scheduling cycle to ensure fairness. Constraints (19) and (20) limit the maximum number of consecutive hours for which the nurses work per day, ensuring that they have sufficient rest to maintain efficiency. Constraint (21) enforces the maximum and minimum number of consecutive working days for each full-time nurse. Constraints (22) and (23) ensure that the logical relations between the decision variables are satisfied, where *M* is a sufficiently large constant. Constraint (24) ensures that no part-time nurses are working during the given time shift. Constraint (25) enforces the minimum and maximum number of days nurses can work during the planning period to ensure reasonable working hours and rest periods. Constraint (26) limits the number of part-time nurses who can work on a given day. Constraints (27) and (28) ensure that the logical relations between decision variables are satisfied, where *M* is a sufficiently large constant. Finally, constraint (29) constrains the decision variables to binary values.

## 3. Results

### 3.1. Data Analysis

**Figure 3:**
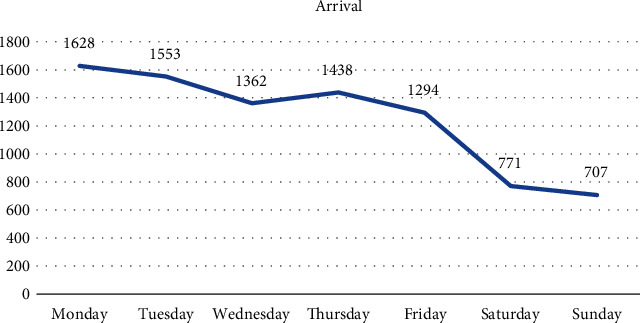
Patients arriving on different days.

Figure 3 shows that different numbers of patients arrive on different days of the week, with peak numbers on Monday and Tuesday and relatively few on Saturday and Sunday. Based on these numbers, we can divide the week into three parts: Monday and Tuesday; Wednesday, Thursday, and Friday; and Saturday and Sunday.  Table 1 shows that the patient arrival rate substantially fluctuates during the day. Hospitals typically prefer to roster a fixed number of staff to work, but such large demand fluctuations can easily lead to one of the two situations: either the staff struggles to meet the demand during peak periods, causing long queues, or they are assigned less tasks during quiet periods when the demand is low, resulting in overstaffing and wasted manpower.

### 3.2. Solution to the Models

The above queuing model is an M/M/C queuing system, which is used herein to solve using R. The proposed algorithm and model are verified using a practical example. This study assumes that patients arrive in the hospital's blood collection hall according to the Poisson process and that the service time is subject to a negative exponential distribution with an average rate of 0.64 minute/person. Based on the hospital requirements quoted above, the example's other main parameters are set to *t*_q_ = 15 min and *P*_q_ = 0.95. Finally, the results of the queuing theory model are calculated using R.

Table 2 shows the minimum number of open blood collection windows during different time shifts. For example, opening nine windows on Monday and Tuesday between 7 a.m. and 10 a.m. is sufficient to meet the demand. The queuing model calculates the probability of any patient's queuing time exceeding the given time limit with a certain number of staff. Most patients have to wait less than 15 min, which is a substantial improvement over the previous situation.

After using the queuing model to calculate the nurses required during each time shift, the following data about hospital A's blood collection nurses were obtained. A total of 15 full-time blood collection nurses and five part-time nurses are routinely available. Each nurse can work for at least three consecutive days, but no more than five days, and can work for up to three time shifts per day. Part-time nurses can only work in the first and second time shifts. Rosters are prepared for four-week periods. Full-time nurses work at least 20 days but less than 24 days. Using these data, we solved the nurse scheduling model.

Clearly, this is an MIP problem and feasible; approximately, optimal solutions are acceptable for nurse scheduling. Here, we used the mature CPLEX solver to solve this MIP problem. By combining the calculated number of blood collection windows that must be opened during each time shift with this example model, CPLEX obtained the most optimal scheduling plan.

According to the optimal solution, a total of 300 person-days are required during the four-week planning period.  Table 3 shows part of the corresponding scheduling plan, including full-time and part-time nurses working during each time shift. For example, during the first period of the first day, full-time nurses 1, 4, 5, 6, 8, 10, 11, and 17 and a part-time nurse must work.  Table 4 shows the days on which each nurse is working during the first week of the 28-day shift period. For example, nurse 1 works on days 1, 2, 5, 6, and 7 during the first week. Tables 3 and 4 show only partial results; for more detailed results, please see Supplementary Materials S1 and S2.

**Figure 4:**
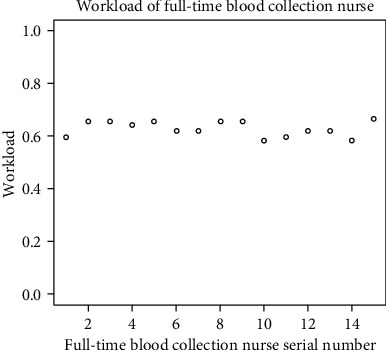
Workloads of the full-time nurses over the planning cycle.

Figure 4 describes each nurse's average workload over the entire planning period (total number of hours worked/total number of time shifts in the planning period) according to the solution. Here, we can see that the nurses' workloads do not considerably fluctuate, implying that the plan is fair.

## 4. Discussion

Based on a systematic analysis of the factors affecting the scheduling of nurses in large hospitals, a flexible scheduling model for blood collection nurses is established using queuing theory and MIP. This combined model can reasonably determine the number of nurses required during particular periods and flexibly arrange rosters while ensuring that they receive reasonable rest periods. The final results of the model denote that the patient's waiting time is effectively alleviated and that a nurse's rest time is ensured.

The large number of constraints in this example and the complexity of the decision variables imply that manual scheduling cannot find optimal solutions. In contrast, the proposed model can rapidly implement nurse scheduling. This flexible scheduling approach can adjust the number of nurses working based on dynamic changes in demand. During periods of low demand, fewer staff is scheduled to work, whereas part-time nurses can be introduced during peak periods to spread the workload. Adapting the model to different hospitals only requires changes to the input data and parameters, enabling managers to easily grasp and promote it. However, several issues pertaining to the proposed model must be addressed. For example, we did not consider an approach to minimizing the hospital's costs when employing part-time nurses or to handling the nurses' leave periods.

In summary, the proposed flexible scheduling model provides an effective solution for rostering nurses in blood centers. In China, hospitals, supermarkets, banks, and government offices struggle with long queues and desire flexible scheduling. The proposed model that is based on queuing theory and MIP could be a flexible scheduling model for such tasks.

## 5. Conclusions

Queuing and nurse scheduling models were proposed herein to solve the issue of rostering nurses. First, the queuing model determined the minimum labor demand during each period of each day, which served as the primary input for the scheduling model. This model was then used to determine the nurses' shift schedules and organize the full-time nurses' shifts to ensure fairness. Results of numerical studies conducted using data from a large hospital in China show a significant improvement in patient waiting time performance metrics over the hospital's current practice. The proposed model could generate reasonable rosters to handle relatively large fluctuations in patient numbers. In summary, we proposed methods for making decisions about staff numbers and scheduling that can improve work efficiency.

## Figures and Tables

**Table 1:** Patient arrival rates (people/min) for different time shifts and days.

Days	7 a.m.–10 a.m.	10 a.m.–1 a.m.	1 p.m.–5 p.m.
Monday, Tuesday	5.23	2.16	0.79
Wednesday, Thursday, Friday	4.56	1.61	0.67
Saturday, Sunday	2.45	0.89	0.42

**Table 2:** Predicted minimum number of open blood collection windows.

Date	*λ*	Period	c.min	*W*_q_	*L*_q_	*P*_q_	*W*	*L*	*P*	*c*_	*u*
Monday	5.23	1	9	1.33	6.98	0	2.9	15.15	0	8.17	0.91
	2.16	2	4	1.69	3.66	0	3.26	7.03	0	3.38	0.84
	0.79	3	2	0.96	0.76	0	2.52	1.99	0	1.23	0.62

Wednesday	4.56	1	8	1.21	5.51	0	2.77	12.63	0	7.12	0.89
	1.61	2	3	2.29	3.69	0.01	3.86	6.21	0.01	2.52	0.84
	0.67	3	2	0.59	0.4	0	2.15	1.44	0	1.05	0.52

Saturday	2.45	1	5	0.65	1.6	0	2.22	5.43	0	3.83	0.77
	0.89	2	2	1.46	1.3	0	3.02	2.69	0	1.39	0.7
	0.42	3	1	2.98	1.25	0.02	4.55	1.91	0.04	0.66	0.66

**Table 3:** Blood collection nurses working during different time shifts.

Date	Period	Nurses	Date	Period	Nurses
1	1	1, 4, 5, 6, 8, 10, 11, 17, 20	15	1	1, 2, 6, 8, 10, 11, 12, 16, 19
1	2	1, 5, 6, 8	15	2	1, 10, 14, 15
1	3	10, 11, 13	15	3	2, 6, 11
2	1	1, 3, 4, 6, 9, 11, 12, 14, 15	16	1	1, 3, 4, 5, 6, 7, 8, 9, 12, 15
2	2	1, 2, 7, 8	16	2	3, 5, 10, 13, 14

**Table 4:** Days that each blood collection nurse works.

Nurse	1	2	3	4	5	6	7	8	9	10
Days worked	1	2	2	1	1	1	2	1	2	1
	2	3	3	2	2	2	3	2	3	3
	5	4	4	4	4	3	5	3	4	4
	6	5	5	5	5	4	6	4	5	5
	7	8	7	6	6	7	7	5	6	7

## Data Availability

The data used to support the findings of this study are restricted by Sichuan Provincial People's Hospital in order to protect patient privacy. Data are available from Sichuan Provincial People's Hospital for researchers who meet the criteria for access to confidential data.

## Supplementary Materials

Supplementary materials S1 and S2 take up too much space; only part of the results is shown in the manuscript (Table 3 and Table 4). Table 3 shows part of the corresponding scheduling plan, including full-time and part-time nurses working during each time shift. Table 4 shows the days on which each nurse is working during the first week of the 28-day shift period.
