# Supplementary material for: Applying Queuing Theory and Mixed Integer Programming to Blood Center Nursing Schedules of a Large Hospital in China
Source: Comput Math Methods Med. 2020 Jul 1;2020:9373942. doi: 10.1155/2020/9373942 (PMC7359737; doi:10.1155/2020/9373942)
Supplement: Supplementary materials — S1 and S2 take up too much space; only part of the results is shown in the manuscript (Table 3 and Table 4). Table 3 shows part of the corresponding scheduling plan, including full-time and part-time nurses working during each time shift. Table 4 shows the days on which each nurse is working during the first week of the 28-day shift period. [file 9373942.f1.pdf]

S1 Blood collection nurses working at each time of day

Date	Period	Nurse number	Date	Period	Nurse number
1	1	1, 4, 5, 6, 8, 10, 11, 17, 20	15	1	1, 2, 6, 8, 10, 11, 12, 16, 19
1	2	1, 5, 6, 8	15	2	1, 10, 14, 15
1	3	10, 11, 13	15	3	2, 6, 11
2	1	1, 3, 4, 6, 9, 11, 12, 14, 15	16	1	1, 3, 4, 5, 6, 7, 8, 9, 12, 15
2	2	1, 2, 7, 8	16	2	3, 5, 10, 13, 14
2	3	5, 11	16	3	1, 13
3	1	2, 3, 7, 9, 11, 15, 16, 18, 20	17	1	1, 2, 3, 4, 6, 7, 8, 9, 12, 13, 14, 15
3	2	3, 6, 10, 13	17	2	2, 4, 5, 8
3	3	8, 10	17	3	5, 11, 14
4	1	3, 6, 8, 9, 10, 11, 12, 15	18	1	1, 3, 4, 6, 7, 8, 10, 11, 13, 14, 15
4	2	2, 4, 5, 13	18	2	1, 3, 4, 6, 7, 8, 10, 11, 13, 14, 15
4	3	10, 14	18	3	12, 13, 14
5	1	2, 3, 4, 7, 8, 10, 11, 12, 14, 16, 17	19	1	3, 4, 5, 8, 9, 11, 14, 15
5	2	5, 7, 9, 15	19	2	3, 7, 13
5	3	1, 10	19	3	4, 12, 15
6	1	4, 5, 7, 12, 13, 15	20	1	5, 6, 7, 11, 13
6	2	9, 14	20	2	1, 2, 3
6	3	1	20	3	10
7	1	3, 4, 5, 8, 10, 13, 14	21	1	8, 10, 12, 14
7	2	6, 7, 8	21	2	1, 4, 6
7	3	1	21	3	2
8	1	2, 4, 6, 7, 9, 15, 16, 17, 18	22	1	4, 5, 6, 7, 8, 9, 12, 13
8	2	6, 16, 17, 18	22	2	2, 3, 5, 11, 11, 15
8	3	9, 15	22	3	1, 6
9	1	3, 5, 9, 10, 11, 12, 16, 18, 19	23	1	3, 6, 8, 9, 11, 13, 16, 18, 19
9	2	1, 3, 16, 18, 19	23	2	6, 10, 15
9	3	2, 15	23	3	10, 11
10	1	6, 9, 10, 11, 12, 14, 16, 18	24	1	4, 7, 8, 9, 10, 11, 12, 14, 15, 16, 12
10	2	7, 17	24	2	1, 2, 3, 5
10	3	10, 11, 15	24	3	10, 11, 13
11	1	1, 2, 6, 10, 11, 12, 16, 17	25	1	2, 4, 9, 10, 11, 12, 14, 15
11	2	2, 3, 7, 8	25	2	1, 5, 13
11	3	1, 5, 15	25	3	8, 11
12	1	2, 4, 5, 8, 10, 13	26	1	1, 3, 7, 9, 10, 11, 12, 15
12	2	1, 6, 9, 12	26	2	5, 13, 14

12	3	3, 7, 14	26	3	2, 4, 6
13	1	2, 3, 5, 9, 12, 13, 15	27	1	1, 2, 6, 7, 8, 12, 14
13	2	1, 4	27	2	3, 4
13	3	14	27	3	5
14	1	3, 4, 10, 11, 13	28	1	5, 6, 9, 10, 11, 15
14	2	4, 11	28	2	1, 2, 14
14	3	7	28	3	7

S2 Blood collection nurse work date

Nurse number	1	2	3	4	5	6	7	8	9	10	11	12	13	14	15	16	17	18	19	20
Date	1	2	2	1	1	1	2	1	2	1	1	2	1	2	2	3	1	3	9	1
	2	3	3	2	2	2	3	2	3	3	2	4	3	4	3	5	5	8	1	3
																			2	
	5	4	4	4	4	3	5	3	4	4	3	5	4	5	4	8	8	9	1	1
																			5	2
	6	5	5	5	5	4	6	4	5	5	4	6	5	6	5	9	1	1	2	
																	0	0	3	
	7	8	7	6	6	7	7	5	6	7	5	9	6	7	6	1	1	2		
																0	1	3		
	1	9	9	7	7	8	8	7	8	9	9	1	7	1	8	1	2			
	1											0		0		1	4			
	1	1	1	8	9	1	1	1	9	1	1	1	9	1	1	1				
	2	1	1			0	0	0		0	0	1		2	0	2				
	1	1	1	1	1	1	1	1	1	1	1	1	1	1	1	1				
	3	2	2	2	1	1	1	1	0	1	1	2	2	3	1	5				
	1	1	1	1	1	1	1	1	1	1	1	1	1	1	1	2				
	5	3	3	3	2	2	2	2	2	2	4	3	3	5	3	1				
	1	1	1	1	1	1	1	1	1	1	1	1	1	1	1	2				
	6	5	4	4	3	5	4	5	3	4	5	5	4	6	5	2				
	1	1	1	1	1	1	1	1	1	1	1	1	1	1	1	2				
	7	7	6	6	6	6	6	6	6	5	7	6	6	7	6	3				
	1	1	1	1	1	1	1	1	1	1	1	1	1	1	1	2				
	8	8	7	7	7	7	7	7	7	6	8	7	7	8	7	4				
	2	2	1	1	1	1	1	1	1	1	1	1	1	1	1					
	0	0	8	8	9	8	8	8	8	8	9	8	8	9	8					
	2	2	1	1	2	2	1	1	1	2	2	1	1	2	1					
	1	1	9	9	0	0	9	9	9	0	0	9	9	1	9					
	2	2	2	2	2	2	2	2	2	2	2	2	2	2	2					
	2	2	0	1	2	1	0	1	2	1	2	1	0	2	2					
	2	2	2	2	2	2	2	2	2	2	2	2	2	2	2					
	4	4	2	2	4	2	2	2	3	3	3	2	2	4	3					
	2	2	2	2	2	2	2	2	2	2	2	2	2	2	2					
	5	5	3	4	5	3	4	3	4	4	4	4	3	5	4					

	2	2	2	2	2	2	2	2	2	2	2	2	2	2	2
	6	6	4	5	6	6	6	4	5	5	5	5	4	6	5
	2	2	2	2	2	2	2	2	2	2	2	2	2	2	2
	7	7	6	6	7	7	7	5	6	6	6	6	5	7	6
	2	2	2	2	2	2	2	2	2	2	2	2	2	2	2
	8	8	7	7	8	8	8	7	8	8	8	7	6	8	8
